# Treatment of Soft Tissue Defects after Minimally Invasive Plate Osteosynthesis in Fractures of the Distal Tibia: Clinical Results after Reverse Sural Artery Flap

**DOI:** 10.3390/medicina59101751

**Published:** 2023-09-30

**Authors:** Jun Young Lee, Hyo Jun Lee, Sung Hoon Yang, Je Hong Ryu, Hyoung Tae Kim, Byung Ho Lee, Sung Hwan Kim, Ho Sung Kim, Young Koo Lee

**Affiliations:** 1 Department of Orthopaedic Surgery, College of Medicine, Chosun University, 365 Pilmundae-ro, Dong-gu, Gwangju 61453, Republic of Korea; leejy88@chosun.ac.kr (J.Y.L.); whogus12@kakao.com (H.J.L.); shyang1234@naver.com (S.H.Y.); ryujh9950@naver.com (J.H.R.); kht2769@naver.com (H.T.K.); 2 Department of Orthopaedic Surgery, Daejung Hospital, 180 Daein-ro, Dong-gu, Gwangju 61473, Republic of Korea; hand8150@naver.com; 3 Department of Orthopaedic Surgery, Soonchunhyang University Hospital Bucheon, 170 Jomaru-ro, Wonmi-gu, Bucheon-si 14584, Republic of Korea; shk9528@naver.com (S.H.K.); nine4141@naver.com (H.S.K.)

**Keywords:** distal tibia fracture, MIPO, reverse sural artery flap

## Abstract

*Introduction*: Distal tibial fractures make up approximately 3% to 10% of all tibial fractures or about 1% of lower extremity fractures. MIPO is an appropriate procedure and method to achieve stable metal plate fixation and osseointegration by minimizing soft tissue damage and vascular integrity at the fracture site. MIPO to the medial tibia during distal tibial fractures induces skin irritation due to the thickness of the metal plate, which causes discomfort and pain on the medial side of the distal leg, and if severe, complications such as infection and skin defect may occur. The reverse sural flap is a well-researched approach for covering defects in the lower third of the leg, ankle, and foot. *Materials and Methods*: Among 151 patients with distal tibia fractures who underwent minimally invasive metal plate fixation, soft tissue was injured due to postoperative complications. We treated 13 cases with necrosis and exposed metal plates by retrograde nasogastric artery flap surgery. For these patients, we collected obligatory patient records, radiological data, and wound photographs of the treatment results and complications of reconstructive surgery. *Results*: In all the cases, flap survival was confirmed at the final outpatient follow-up. The exposed area of the metal plate was well coated, and there was no plate failure due to complete necrosis. Three out of four women complained of aesthetic dissatisfaction because the volume of the tunnel through which the skin mirror passed and the skin plate itself were thick. In two cases, defatting was performed to reduce the thickness of the plate while removing the metal plate. *Conclusions*: Metal plate exposure after distal tibial fractures have been treated with minimally invasive metal plate fusion and can be successfully treated with retrograde nasogastric artery flaps, and several surgical techniques are used during flap surgery.

## 1. Introduction

Distal tibial fractures make up approximately 3% to 10% of all tibial fractures or about 1% of lower extremity fractures. In 70% to 85% of cases, there is also a concomitant fibular fracture, indicating more complex injuries [1]. It is a break in the lower part of the shinbone that includes the metaphyseal region and can potentially extend to the weight-bearing joint surface. It is also referred to as a tibial pilon fracture or tibial plafond fracture when the joint surface is involved [2]. Intra-articular fractures occurring at the distal end of the tibia, which involve a substantial portion of the weight-bearing articular surface, present formidable complexities and difficulties in their therapeutic management [3]. Distal tibial fractures are caused by high-energy shear and rotational forces [4] and have a high probability of complications such as delayed union, nonunion, joint stiffness, and soft tissue necrosis due to the small amount of surrounding soft tissue, poor blood flow, and thin periosteum. Fractures located at the distal tibia typically necessitate surgical intervention, as non-operative approaches involving lengthy application of long leg casts, extended immobilization, and the inherent risk of malunion are less favored. Non-operative treatment is predominantly reserved for individuals with significant comorbidities that might hinder their ability to undergo anesthesia, as well as for patients with minimally displaced fractures [5].

The most appropriate treatment for distal tibial fractures is a subject of controversy [6]. Surgical treatment methods include open reduction, internal fixation using a metal plate, intramedullary nailing, minimally invasive plate osteosynthesis (MIPO), and external fixation [7,8]. Over the past six decades, there has been remarkable and rapid growth in the field of surgical techniques and understanding of fracture treatment. This progress can be highlighted by comparing Robert Danis’ original principles of osteosynthesis to the current principles advocated by the AO (Arbeitsgemeinschaft für Osteosynthesefragen). While Danis emphasized the precise anatomic restoration of bones and achieving absolute stability, the contemporary theory has evolved to allow for the restoration of anatomic relationships (such as length, alignment, and rotation), even if not directly at the articular surfaces. Current approaches also accept the concept of relative stability, promoting callus formation and giving importance to cautious soft tissue handling. These differences clearly distinguish traditional and minimally invasive plating techniques, signifying the advancements that have taken place in fracture management over the years [9]. The primary goal of surgical intervention is to rectify the anatomical positioning of the lowermost tibia and furnish adequate stability, thereby fostering the mending of fractures and reducing delayed complications [10].

Extensive scholarly material exists concerning the management of fractures situated along the tibial diaphysis, and intramedullary nailing (IMN) is commonly characterized as the foremost therapeutic option for the vast majority of cases [11]. Intramedullary nailing (IMN) offers the advantage of being minimally invasive, leading to smaller skin incisions, reduced soft-tissue trauma, and preservation of blood supply around the bone. The fixation achieved through IMN is stable, allowing for early mobilization. However, there is a possibility of experiencing anterior knee pain, and malunions have been reported in cases of distal tibial fractures treated with this method [5]. Inserting the nail tip within the uppermost third between the tibial plateau and tibial tuberosity, and exhibiting nail prominence of over 5 mm beyond the front surface of the tibial cortex, emerged as variables correlated with post-intramedullary nailing (IMN) knee discomfort following tibial diaphyseal fractures [11]. While intramedullary fixation offers benefits such as better preservation of soft tissue and blood supply, the management of fracture fragments becomes increasingly difficult as the fracture approaches the articular surface, transitioning from the diaphysis to the metaphysis. As the gap between the intramedullary nail and the cortex widens, the likelihood of malreduction and malunion also increases [9]. Within the central region of the tibial shaft, IMN may enable adequate alignment, yet this length remains abbreviated. The upper or lower portion of the shaft presents variability, precluding IMNs from interacting with the tibial cortex in these regions. In such cases, sustaining the alignment relies exclusively on anchoring the locking nails at the proximal or distal area. As a result, the IMN fixation manifests relative fragility, leading to limited torsional resilience [12].

On the other hand, open reduction and internal fixation (ORIF) carry a lower risk of malunion but require a longer time before weight-bearing is allowed and pose an increased risk of wound complications [5]. ORIFs were utilized to restore the anatomical integrity of the joint surface. Spiral oblique and spiral wedge fractures arise from different mechanisms than transverse fractures. These fractures materialize due to rotational forces, and such rotational dynamics can engender extensive fracture patterns extending toward metaphyseal regions. Plates exhibit superior resistance against torsional forces in comparison to intramedullary nails (IMNs), rendering them a potentially more fitting selection for managing spiral oblique and spiral wedge fractures [12]. Nevertheless, the extensive dissection of soft tissue resulted in elevated infection rates and complications related to the soft tissue. Furthermore, it has been observed that open reduction and plate fixation can modify the blood supply to the tibia, potentially leading to delayed union or nonunion [3]. Using external fixators in the management of high-energy distal tibia fractures provides the benefit of significantly reducing soft tissue dissection and minimizing disruption of the blood supply. This proves advantageous in cases where there is extensive soft tissue damage and traumatized skin. Moreover, high-energy distal tibia fractures are frequently associated with other bodily trauma. Therefore, the temporary application of an external fixation device allows for additional time to attain hemodynamic stability and address other life-threatening injuries [13]. 

MIPO is a procedure that accounts for the biomechanics of fractures and is an appropriate method for achieving stable metal plate fixation and osseointegration by minimizing soft tissue damage and vascular integrity at the fracture site using an indirect reduction technique [14]. The MIPO technique offers clear advantages, such as reduced soft tissue damage and improved osteointegration; when compared to conventional surgical methods, it still faces several significant issues that remain unresolved. In research conducted by Hasenboehler et al., 32 patients with distal tibial fractures were subjected to minimally invasive plate osteosynthesis (MIPO). The study revealed a tendency for delayed union in cases of simple fracture patterns. Despite the advantage of preserving the blood supply, MIPO does not facilitate optimal fracture reduction. In simple fractures, the fragments may not be anatomically aligned, and interfragmentary compression might be inadequate, leading to delayed union. Furthermore, there have been reports of other intraoperative complications, including damage to the saphenous nerve and vein [7]. In addition, MIPO to the medial tibia during distal tibial fractures induces skin irritation due to the thickness of the metal plate, which causes discomfort and pain on the medial side of the distal leg, and if severe, complications such as infection and skin defect may occur. According to T.W. Lau et al., of a total of 48 patients who underwent MIPO after distal tibia fracture, infection occurred in 7 patients (15%) and skin irritation due to exposed metal plates was reported in 25 patients (52%) [15]. Treating postoperative soft-tissue defects on the lower legs presents significant challenges due to arterial and venous insufficiency, compromised skin quality characterized by epidermal and dermal atrophy, limited tissue laxity, and an elevated risk of infection [16]. Due to this anatomical characteristic and frequent bone exposure, most surgeons have come to regard free flaps as the primary treatment option [17]. Microvascular flaps are outstanding alternatives, but their surgical procedure is challenging and demands a skilled team, advanced equipment, and specialized hospital centers. Cutaneous and fasciocutaneous flaps with a distal pedicle are another viable option to be taken into consideration. The reverse sural flap is a well-researched approach for covering defects in the lower third of the leg, ankle, and foot. It relies on the communicating and perforating branches of the fibular artery, originating approximately 5 to 6 cm cranially to the lateral malleolus [18]. 

Therefore, the present authors performed retrograde superficial sural artery flap surgery for the distal metal plate exposure, which occurred as a complication after MIPO for distal tibial fracture. The aim of this study is to suggest some surgical tips with a small cohort to improve the outcome and success rate after MIPO for distal tibia fracture.

## 2. Materials and Methods

From June 2010 to December 2020, among 151 patients with distal tibia fractures who underwent MIPO at our hospital, 13 patients (9%) had soft tissue injury due to postoperative necrosis. We treated 13 cases with necrosis and exposed metal plates by retrograde superficial sural artery flap surgery, and for these patients, we collected patients’ hospital records, radiological data, and wound photographs of the treatment results and complications of reconstructive surgery. A retrospective analysis was originally performed, and patients who could be followed up for at least 12 months after surgery were included (Table 1). The female-to-male ratio was 44.4%.

### Surgical Technique

Initially, the patient was placed in a prone position under spinal or general anesthesia, and the pneumatic tourniquet was at 300 mmHg. Then, debridement on the metal plate exposed area was performed. A circular or oval shape was drawn with a marking pen along the area of the skin, which is considered normal around the visible metal plate exposed area, and then excised with a scalpel. In order to suture the sural artery flap, a relatively thick pedicle flap containing subcutaneous fat and fascia, skin-to-skin without tension at the defect, even if the exposed area is small during excision, the defect is at least 4 × 3 cm^2^ in size, which was enlarged and excised.

The axis of the flap draws an imaginary line connecting the center of the back of the lower leg from the point approximately 3–5 cm proximal to the lateral malleolus of the ankle joint to the popliteal fossa, considering the travel of the small saphenous vein and the peroneal artery. A donor flap was drawn on this line.

The pivot point of the arc of rotation is set at a point approximately 8–10 cm proximal in the lateral malleolus of ankle joint surgery to maximize preservation of the perforator from the peroneal artery, maintain arterial blood flow, and make it possible to rotate the flap without twisting to the defect in the ankle joint area. 

Next, the length of the skin mirror was measured with a tape along the movement path of the skin mirror from the reference point of the rotation arc to the metal plate exposed area, and about 1 cm was added to this to determine the length of the skin mirror. The shape of the flap was circular or oval, depending on the shape of the defect, and the distal portion of the flap was shaped like a teardrop with a length of about 2 cm. When the skin was sutured in the defect, the inlet of the skin mirror was cut in a straight line of the same size and sutured with the distal flap to reduce the pressure on the skin mirror. The size of the flap was set to be about 0.5 cm larger than the defect in men and about 1 cm larger in diameter than the defect in women with relatively large amounts of subcutaneous fat.

The elevation of the flap proceeded from the proximal part to the distal part, and the dissection between the fascial layer and the muscular layer was bluntly dissected with a Hemostat, etc., and the distal part was elevated. In this process, the peroneal nerve was preserved as much as possible if possible, and when the peroneal nerve entered the upper fascial layer, it was cut. In this study, the peroneal nerve was preserved without cutting in 5 cases. After lifting the flap to the center of rotation, the flap was moved to the defect, and when it was determined that the flap could be moved without tension, the dissection of the fascia and muscular layer was stopped.

Afterwards, the pressure tourniquet was released to ensure the flap had good blood circulation, and the suture aligned the transferred flap skin-to-skin with the skin of the defect. The donor site, considering the size and condition of the site, was sutured, and the procedure was completed after placing a drainage tube to prevent complications such as venous congestion. The operation site was covered with a transparent cover to check for complications, such as postoperative necrosis around the flap suture, and the flap was protected with splint fixation to strictly limit the range of motion of the ankle joint and bearing of weight.

## 3. Results

In all cases, flap survival was confirmed at the final outpatient follow-up. The exposed area of the metal plate was well coated, and there was no plate failure due to complete necrosis. In one case (8%), there were color changes and slight blister formation due to venous congestion, but they survived without partial necrosis. Venous congestion and marginal skin necrosis were found in the other case; however, it was healed by secondary healing after antibiotics and daily dressing of the flap wound site, and no additional surgical treatment was required. Additionally, patients with sural nerve damage immediately after surgery had side effects such as hypoesthesia and numbness of the lateral leg in one case (8%); however, they gradually adapted over time, and there was no occurrence of a neuroma.

Three out of four women complained of aesthetic dissatisfaction because the volume of the tunnel through which the skin mirror passed and the skin plate itself were thick. In two cases, defatting was performed to reduce the thickness of the plate while removing the metal plate.

**Case 1.**

**Figure 1:**
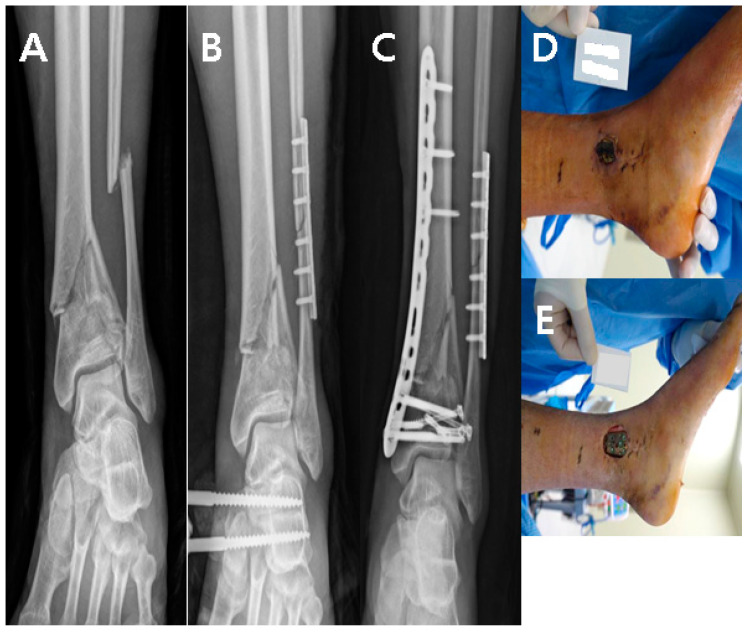
(**A**) Pilon fracture by 2 m F/D. (**B**) For the two-stage operation, EF was done. (**C**) Conversion operation was performed using the MIPO technique. (**D**,**E**) One month after MIPO, skin defect and plate exposure occurred.

**Figure 2:**
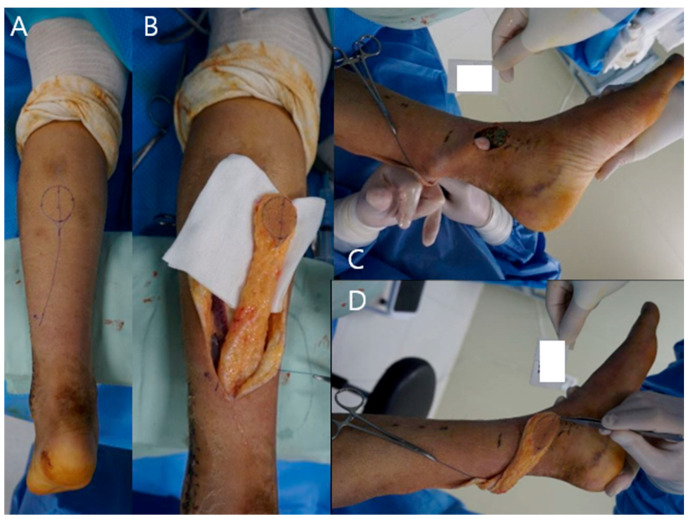
(**A**) A flap on the donor site was designed. (**B**) Drawing the line from the popliteal fossa to the lateral malleolus; the pivot point was set to 10 cm proximal to approximate the vascular axis of the flap. (**C**,**D**) The flap was passed through the subcutaneous tunnel to the defect site.

A 22-year-old man visited our emergency center with a pilon fracture caused by a 2 m fall down accident. Radiological examination revealed severe shortening of the tibial joint surface, and emergency surgical treatment using external fixation was performed. Two weeks later, conversion using the MIPO technique for tibia and plate fixation in the fibula was performed (Figure 1A–C). One month after internal fixation, soft tissue defects and metal plate exposure were observed on the medial side of the left ankle (Figure 1D,E).; therefore, a reverse sural artery flap was performed (Figure 2A–D). The size of the flap was 2 × 3 cm^2,^ and the length of the flap pedicle was 7 cm. The donor site was sutured, and the donor site and flap survived without complications at the final outpatient follow-up.

**Case 2.**

**Figure 3:**
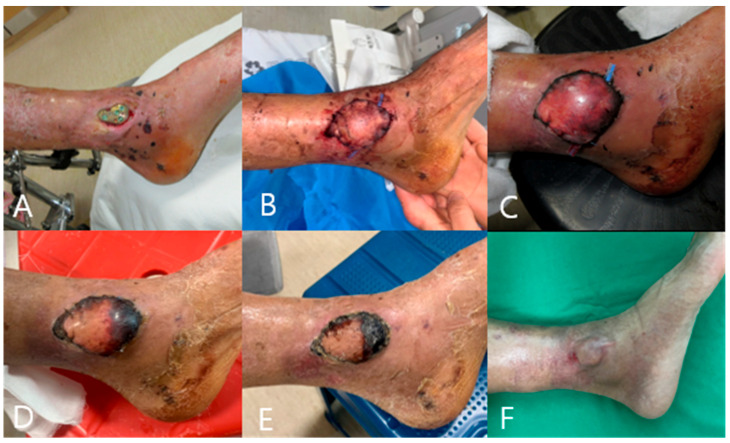
A 64-year-old man with a distal tibia extra-articular fracture by a car accident. (**A**) The wound before doing reverse sural artery flap. (**B**–**D**) After reversing the sural artery flap, venous congestion and marginal skin necrosis occurred. (**E**,**F**) Using antibiotics and dressing daily, the flap site wound healed by secondary healing.

A 64-year-old man visited our emergency center with a closed distal tibial fracture caused by a pedestrian TA. At the time of the injury, the swelling around the ankle joint was severe, and there were many bulla in the soft tissue and abrasions. Therefore, internal fixation was performed using the MIPO technique 3 weeks after the injury. In the subsequent wound management process, signs of infection, accompanied by redness, heat, and pain on the medial side of the shin and soft tissue defects, were observed (Figure 3A). Bacterial identification tests on the wound area confirmed the presence of GNR. Under antibiotic maintenance, the wound was in good condition, and a reverse sural artery flap was performed (Figure 3B,C). The size of the flap was 4 × 4 cm^2,^ and the length of the flap pedicle was 9 cm. Postoperatively, venous congestion around the flap and marginal skin necrosis were observed (Figure 3D,E). Additional surgical treatment was considered; however, the flap site recovered through secondary healing using antibiotics and daily dressing (Figure 3F).

## 4. Discussion

**Figure 4:**
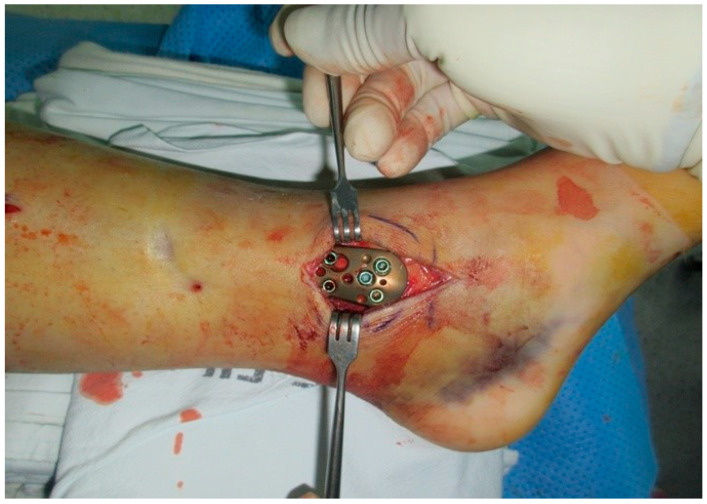
Minimally Invasive Plate Osteosynthesis (MIPO) procedures.

Distal tibial fractures have a high risk of complications such as infection and soft tissue defects due to their complex fracture pattern and the small amount of soft tissue around the ankle joint [19], making it challenging for surgeons to select an appropriate treatment method. Owing to its biomechanical advantage of minimizing damage to the injured soft tissue and maintaining the vascular integrity of the periosteum, MIPO is one of the preferred procedures for treating distal tibial fractures [20,21,22]. However, when a metal plate is inserted into the inside of the ankle joint, the metal plate and the bone often float without being in close contact with each other, which can cause discomfort and pain (Figure 4). Due to this, skin irritation of the ankle causes soft tissue defects, resulting in complications such as metal plate exposure. The present authors also experienced such soft tissue defects. When a soft tissue defect occurs in which the metal plate is exposed in the distal tibia, the operator has no choice but to suffer embarrassing complications. When the metal plate is exposed at the distal tibia, primary suturing is almost impossible, no matter how small the defect is. It is also not easy to cover the defect by using regional flap or muscle flap transposition. 

In this study, a reverse sural artery flap was used, which has been proven to be clinically effective in treating defects in the distal lower extremities, such as the tibia ankle and heel defects [23,24]. Similar to the results of this study, several studies have shown good results with the procedure in the lower limbs [25,26,27]. This procedure is a myofascial skin flap based on the sural artery network; therefore, unlike the free flap, it does not require microsurgery using a microscope, making the procedure simple. Moreover, it provides stable blood flow to the flap without damaging major arteries of the lower leg, and this whole procedure can be performed by a single operator [28,29,30]. The authors attempted to suggest some surgical methods to consider during surgical treatment, along with the results of the patient treatment outcomes.

First, the skin plate size must be determined by performing a marginectomy on the exposed metal plate. The size of the defect after the procedure was larger than before the marginectomy. We kept the defect size to be at least 4 × 3 cm ^2^ after marginectomy. It was for the sural artery flap (comparatively thick pedicle flap containing subcutaneous fat and fascia), which is being sutured skin-to-skin without tension. The width of the flap diameter is a minimum of 4 cm; it was thought that if it was smaller than this, it would be difficult to place the flap on the defect without tension.

The superficial sural artery originates from the proximal popliteal artery anastomoses with the venous and neuroskin perforators of the peroneal artery approximately 3–5 cm proximal to the lateral malleolus. Through this anastomosis, retrograde blood flows back into the sural artery. These venous skin perforators play a critical role in the blood circulation of the flap [24,31,32]. Considering this, when performing reverse sural artery flap, we set a pivot point approximately 3–5 cm proximal to the last venous and neuroskin perforators. However, the authors set 8–10 cm points for saving the perforated area that is as close to the plate as possible, and the plate can be applied to the more proximal part, near the center of the back of the inferior part, and the donor part can be completed through primary suturing without partial or full-thickness skin grafting. This was to allow the plate to move to the defect without twisting the plate-receiving mirror. The peroneal artery runs along the fascia, and therefore, fascia must be included to facilitate the supply of the retrograde reverse sural artery flap. The suprafascial network plays an especially important role, and venous drainage is accomplished by a connection between the subcutaneous lesser saphenous vein and the deep-lying Venae comitantes of the sural nerve. 

In contrast, the reverse sural artery flap is known to have some disadvantages, such as venous congestion, necrosis of the soft tissue around the flap suture, and neuroma from sural nerve damage. According to Hasegawa et al., venous congestion is a common complication of the reverse sural artery flap, which is caused by the presence of valves in the deep venous system that obstructs retrograde venous blood flow. If the flap is not elevated with the fascia during the operation, soft tissue necrosis around the flap suture site may occur [31]. To prevent such complications, several studies have reported that pedicle width plays an important role in flap survival. Sugg et al. recommended a pedicle width of a minimum of 4 cm to preserve the venous drainage and Colossus at the Adipofascial skin plate containing subcutaneous tissue on the skin mirror and improve flap survival rate during reverse sural artery flap [33]. Considering that the thin, soft tissue and flap of the distal tibial defect must pass through the subcutaneous tunnel to the defect, the authors used a 3 cm width of the subcutaneous tissue for the flap pedicle and an inferior width of the fascia of the skin is 5 cm. This method reduces the volume of the flap diameter, facilitates passage of the flap through the tunnel and, at the same time, secures the stability of blood circulation. The fascia part of the plate was also 1 cm wider than the skin and made a colossus. 

When making a subcutaneous tunnel through which the flap pedicle passes, it is expanded wide enough to pass two fingers so that the flap pedicle is not pushed. The distal part of the flap was set in a teardrop shape, and a 2 cm incision was made at the proximal part of the connection line so that the flap can be sutured without pressure and tension when the flap and the skin of the defect are sutured.

The authors found that the flap survived in the previous case, partial necrosis along the skin suture boundary occurred in one case, which healed with the use of prophylactic antibiotics and dressings alone, and venous congestion occurred in one case from the day after surgery. Discoloration occurred but healed well with Colossus alone without the use of medical leeches. In the authors’ case, the better results compared to other studies were flap coverage performed in the same setting of plate exposure occurring after minimally invasive metal plate fixation, and the size of the flap was relatively small. In addition to the fact that it was in the site, it is thought that the fact that the authors designed and colossalized the flaps and provided technical modifications also helped.

This is thought to be caused by insufficient dissection of the flap, which left out some fascia during flap elevation. Each operator performs reverse sural artery flap surgery in a slightly different manner, depending on the patient’s clinical experience. If the sural nerve is cut during surgery, the patient complains of muscle weakness in the ankle, paresthesia, or pain in the distal lower extremities. According to Touam et al., there are sometimes cases that require additional surgical treatment due to the development of neuroma [34]. The authors sought to preserve possible affected peroneal nerves during flap coverage. However, in the remaining cases, the peroneal nerve migrated to the upper fascia relatively early during flap colossus, necessitating fasciotomy for nerve preservation, and nerve preservation was abandoned due to concerns about flap blood circulation and disconnected. A nerve amputation patient had numbness and cramping in the lateral foot, but this patient appeared to improve over time and did not develop a nerve amputation neuroma.

The limitations of this study are as follows: First, given that the number of cases was small and this was a retrospective study based on medical records and radiological data, more studies based on a larger number of cases are needed to confirm that the reverse sural artery flap is indeed an effective treatment for patients who underwent internal fixation through the MIPO procedure. The fact that this study did not mention a correlation with the patient’s underlying disease, such as the presence of vascular diseases that may affect the survival rate of the flap, should also be supplemented in future studies. 

However, for the treatment of metal plate exposures that occur after MIPO procedures for peripheral tibial fractures, retrograde superficial sural artery flaps may be an effective treatment. This study is meaningful in that it suggested a surgical technique which could improve outcomes and success rates.

## 5. Conclusions

Metal plate exposure treatments that occur after distal tibial fractures that have been treated with the MIPO procedure can be successfully treated with a retrograde superficial sural artery flap, and it is believed that more successful flap survival can be achieved by applying several surgical techniques during the flap.

## Figures and Tables

**Table 1:** Patients Demographics.

	Age/Sex	Injury Mechanism	MIPO Op. interval (Days)	MIPO to RSSA Op. interval (mo.)	Wound Culture
**1**	49/F	IncarTA	7	0.5	No growth
**2**	28/M	Sports	8	1.5	No growth
**3**	59/M	IncarTA	4	1.3	No growth
**4**	64/M	Fall down	21	0.6	**GNR**
**5**	81/M	IncarTA	9	1.1	**GPC**
**6**	63/F	Fall down	11	0.8	No growth
**7**	52/M	Sports	13	1.4	**GPC**
**8**	79/M	Fall down	2	1.0	No growth
**9**	37/F	IncarTA	6	0.4	No growth
**10**	22/M	IncarTA	14	0.9	No growth
**11**	77/F	Fall down	10	1.1	No growth
**12**	56/M	Sports	8	1.5	No growth
**13**	23/M	Fall down	6	1.6	No growth

TA: traffic accident. GNR: Gram-negative bacillus. GPC: Gram-positive coccus.

## Data Availability

Data sharing is not applicable to this article because there were no datasets made or analyzed during this study.
